# Crucial role of stimulator of interferon genes-dependent signaling in house dust mite extract-induced IgE production

**DOI:** 10.1038/s41598-021-92561-w

**Published:** 2021-06-23

**Authors:** Hiroki Nunokawa, Yusuke Murakami, Takashi Ishii, Tomoya Narita, Haruyuki Ishii, Hajime Takizawa, Naomi Yamashita

**Affiliations:** 1 Department of Pharmacotherapy, Research Institute of Pharmaceutical Sciences, Musashino University, 1-1-20 Shinmachi, Nishitokyo, Tokyo 202-8585 Japan; 2 Department of Respiratory Medicine, Kyorin University School of Medicine, Mitaka, Tokyo Japan; 3 Department of Pharmaceutical Sciences, Faculty of Pharmacy, Musashino University, Nishitokyo, Tokyo Japan

**Keywords:** Cell biology, Immunology, Diseases

## Abstract

Stimulator of interferon genes (STING) is a DNA sensor that responds to pathogens and induces type I interferon production. Herein, the role of STING in house dust mite extract (HDM)-induced allergic asthma was investigated. C57BL/6 wild-type (WT) and *Sting*^−/−^ mice were intratracheally sensitized with HDM, and the bronchoalveolar lavage fluid (BALF), sera, lungs, and mediastinal lymph nodes (MLNs) were analyzed. The total and HDM-specific serum IgE levels were lower in *Sting*^−/−^ mice than in WT mice. B cell and IgE-positive B cell proportion in BALF and MLNs, respectively, was significantly lower in *Sting*^*−/−*^ mice than in WT mice. Additionally, cyclic GMP-AMP, a STING ligand, augmented total and HDM-specific serum IgE levels and B cell proportion in BALF when applied in combination with HDM. To elucidate the role of STING in IgE production, follicular helper T (Tfh) cells, which are involved in B cell maturation, were investigated. Tfh cell proportion in MLNs decreased in *Sting*^*−/−*^ mice, and IL-4 and IL-13 production by HDM-restimulated MLN cells from HDM-sensitized mice was decreased in *Sting*^*−/−*^ mice compared with WT mice. Thus, STING plays an important role in the maturation and class switching of IgE-producing B cells in allergic inflammation via Tfh cells.

## Introduction

Stimulator of interferon genes (STING) is a transmembrane protein localized in the endoplasmic reticulum. In 2008, it has reported as one of the most important factors involved in innate immunity via the production of type I interferons^1^. The pathway is initiated when exogenous DNA, such as viral and bacterial DNA, binds to cyclic GMP-AMP synthase (cGAS) in the cytoplasm^2,3^. Activated cGAS synthesizes cyclic GMP-AMP (cGAMP) as a second messenger from GTP and ATP. STING activated by cGAMP induces phosphorylation of IRF3, which produces type I interferons^4–6^. It has also been reported that cyclic dinucleotide leaking from bacteria directly activates STING*.* In addition, STING is activated by endogenous DNA, such as that from dead cells and damaged mitochondria, and it induces the expression of inflammatory cytokines, such as tumor necrosis factor-α and interleukin (IL)-6^7^. STING plays a significant role in inflammatory diseases and infections^8–12^. Inflammatory response is a topic of controversy in studies related to the murine systemic lupus erythematosus (SLE) model. While one study has showed an improvement in inflammatory response^13^, another reported that it had worsen^14^ in the presence of STING. Therefore, the mechanism through which STING is involved in inflammatory response is presently unclear^15^.

Allergic asthma is a chronic inflammatory disease characterized by reversible airway obstruction, airway inflammation, and airway hypersensitivity. This chronic inflammation is initiated by IgE cross-linking on mast cells and basophils, following the release of chemical mediators, such as histamine and leukotriene, causing airway smooth muscle contraction, airway hyperresponsiveness, and chronic eosinophil inflammation^16,17^. Although adaptive immunity plays a role in inducing IgE production and airway inflammation, innate immunity has also been reported to play an important role^18–21^. House dust extract mite (HDM), major cause of allergic asthma, activate *s*ignaling through Toll-like receptor (TLR) 4 and TLR2 and is involved in asthmatic inflammation^22–25^. In addition to the fact that group 2 mite allergens have functional homology with a critical molecule (myeloid differential factor 2) in the signal transduction of TLR4, group 2 mite allergens can stimulate epithelial cells through TLR2^24,26^. Furthermore, DNA derived from infiltrating neutrophils is present in allergic inflammatory lesions in humans and mice^27–30^. Although DNA is expected to be involved in asthma, only a few studies have reported the use of DNA sensors in allergic diseases. We previously have reported that TLR9-mediated response to recognize single-stranded DNA exacerbates allergic asthma in a murine HDM-induced asthmatic model^31^. McKee et al. has reported that host DNA released in response to an aluminum adjuvant enhances allergic inflammation, suggesting the partial involvement of STING in the response^32^. In addition, allergic inflammation is increased by cGAMP, a ligand of STING, in a mouse model of HDM-induced allergic asthma^33^. Although the current study suggests the involvement of STING in the pathophysiology of asthma, the precise mechanisms remain unknown. In this study, we attempted to clarify the involvement of STING in asthma using a *Sting*^*−/−*^ HDM-induced asthmatic mouse model.

## Results

### Lack of difference in the number of cells in BALF between ***Sting***^***−/−***^ and WT mice

**Figure 1:**
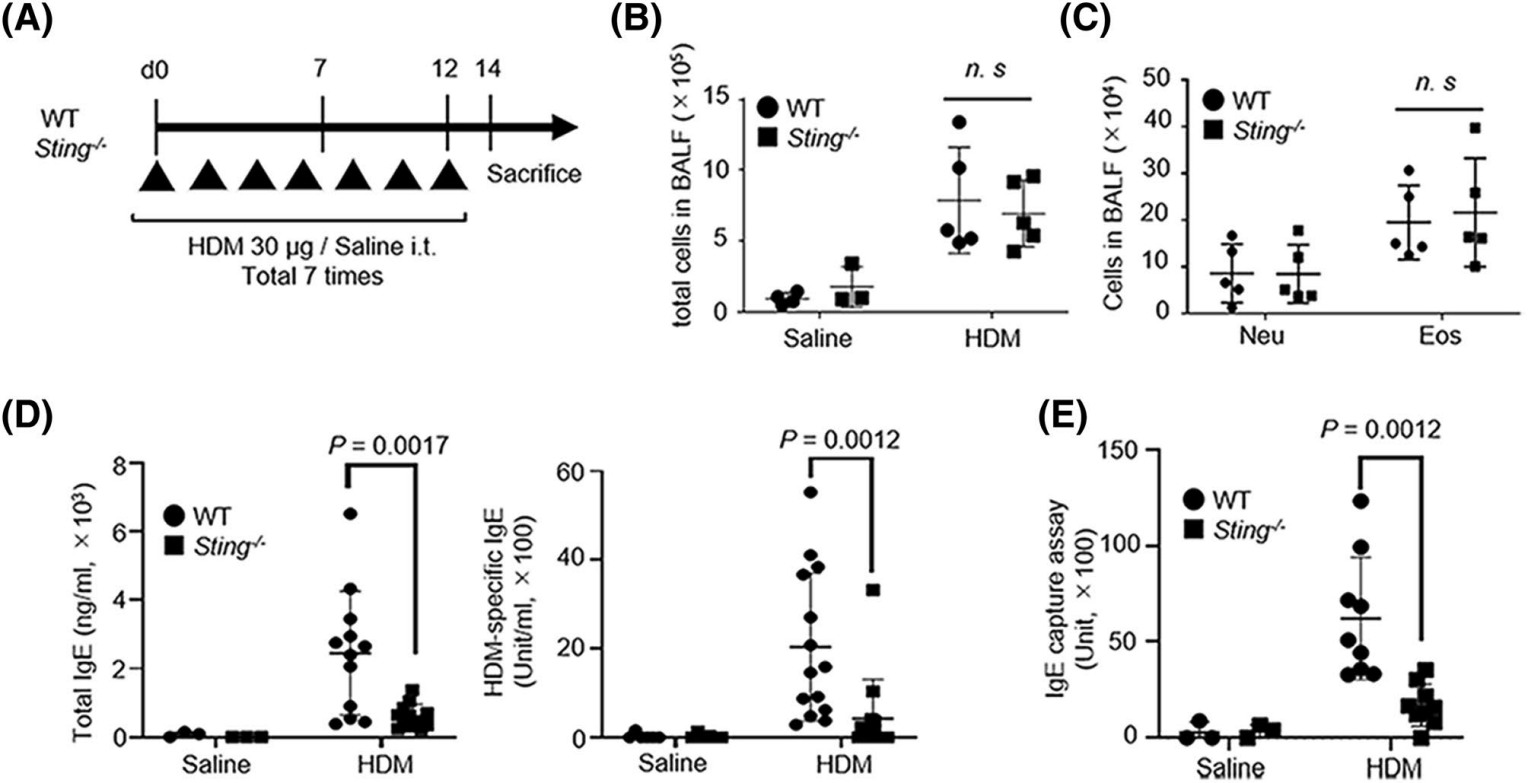
Assessment of HDM-induced allergic asthma in STING-deficient mice. (**A**) Strategy for establishing the HDM-induced allergic asthma model. Wild-type (WT) or *Sting*^*−/−*^ mice were intratracheally administered HDM at a concentration of 30 µg/mouse seven times every other day. The mice were sacrificed 48 h after the last dose. Saline served as a control. (**B**) Total cell counts in bronchoalveolar lavage fluid (BALF) of WT and *Sting*^*−/−*^ mice. Saline-treated WT mice: N = 4, *Sting*^*−/−*^ mice: N = 3, HDM-sensitized WT mice: N = 5, *Sting*^*−/−*^ mice: N = 5. (**C**) The number of neutrophils and eosinophils in BALF of HDM-sensitized WT and *Sting*^*−/−*^ mice. HDM-sensitized WT mice: N = 5, *Sting*^*−/−*^ mice: N = 5. (**D**) Total (left) and HDM-specific (right) IgE levels in the serum from the indicated mice. Saline-treated WT mice: N = 3, *Sting*^*−/−*^ mice: N = 3, HDM-sensitized WT mice: N = 12, and *Sting*^*−/−*^ mice: N = 12 for total IgE levels. Saline-treated WT mice: N = 6, *Sting*^*−/−*^ mice: N = 6, HDM-sensitized WT mice: N = 14, and *Sting*^*−/−*^ mice: N = 14 for HDM-specific IgE levels. (**E**) Serum IgE capture assay in the indicated mice. Saline-treated WT mice: N = 3, *Sting*^*−/−*^ mice: N = 3, HDM-sensitized WT mice: N = 9, and *Sting*^*−/−*^ mice: N = 9. All data are presented as the mean ± SD and (**A**–**C**) data are representative of four or five independent experiments. (**D**) Total IgE levels are representative of three independent experiments. HDM-specific IgE levels are representative of six independent experiments. (**E**) the data are representative of six independent experiments. *i.t*. intratracheally, *n.s* not significant, *Neu* neutrophils, *Eo* eosinophils.

First, we examined the total number of cells in the BALF of allergic asthma model mice. Female B6 WT and *Sting*^/−^ mice were intratracheally administered HDM extract (Fig. 1A). Total cell, eosinophil, and neutrophil numbers in BALF were higher in the HDM group than in the saline group (Fig. 1B,C). However, when the HDM-treated WT and *Sting*^−/−^ mouse groups were compared, no significant differences were observed. The expressions of STING were confirmed in lung of WT but not *Sting*^*−/−*^ mice (see Supplementary Fig. 1).

### Reduction in serum IgE levels and the number of B cells in BALF of HDM-treated ***Sting***^−/−^ mice

Next, we measured the total IgE and HDM-specific IgE levels in the serum using enzyme-linked immunosorbent assay (ELISA). In the HDM group, both total IgE and HDM-specific IgE production significantly decreased in *Sting*^−/−^ mice compared with WT mice (Fig. 1D). Furthermore, we confirmed HDM-specific IgE production using IgE capture assay to exclude the influence of IgG blocking antibody. As shown in Fig. 1E, IgE units decreased in *Sting*^−/−^ mice compared with WT mice in the HDM group.

**Figure 2:**
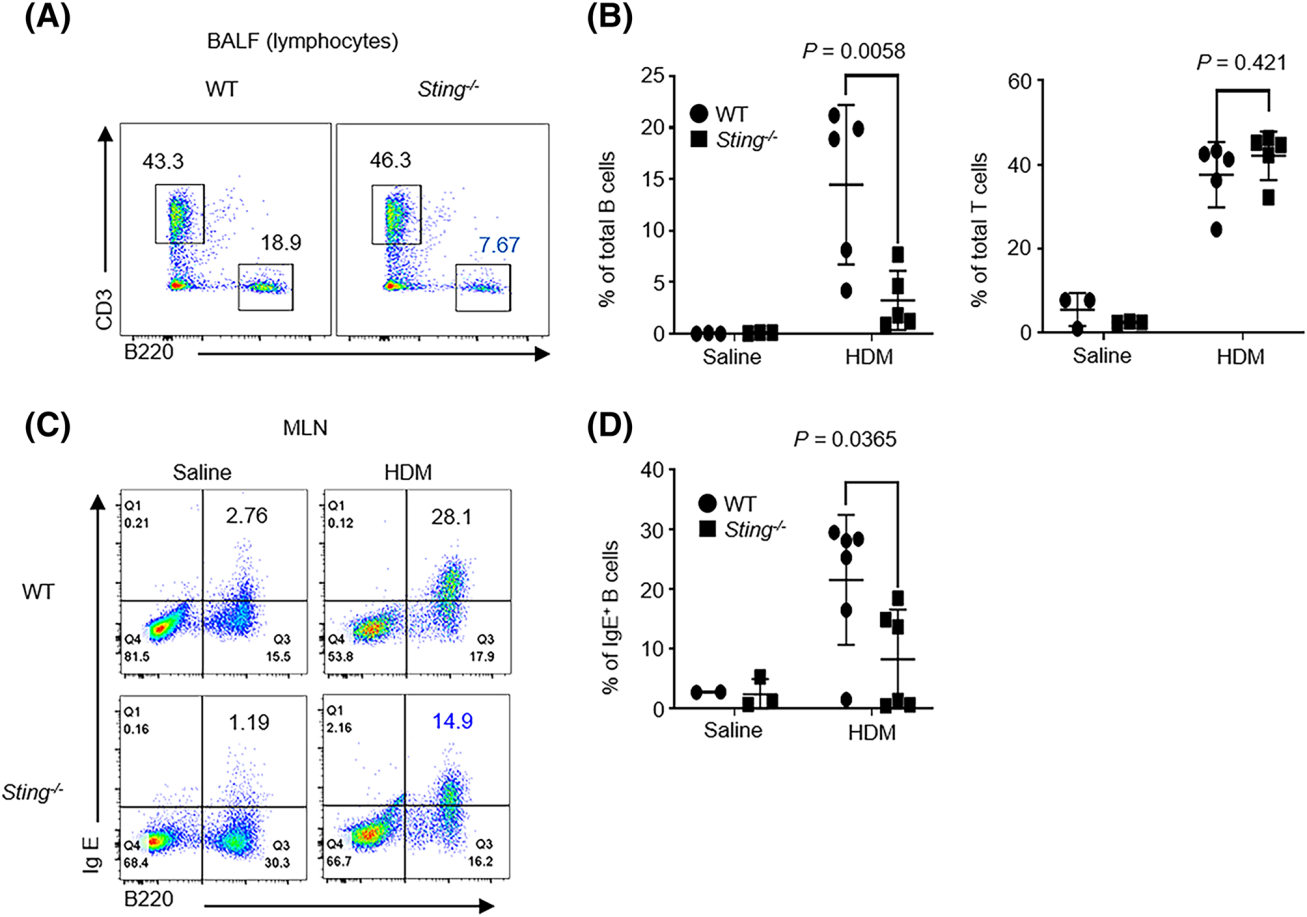
Percentage of lung B cells and serum IgE levels reduced in HDM-sensitized *Sting*^*−/−*^ mice. (**A**,**B**) The percentage of B cells (B220+) and T cells (CD3+) in BALF of HDM-sensitized WT or *Sting*^*−/−*^ mice. Saline-treated WT mice: N = 3, *Sting*^*−/−*^ mice: N = 3, HDM-sensitized WT mice: N = 5, and *Sting*^*−/−*^ mice: N = 5. (**C**,**D**) The percentage of IgE-positive B cells (IgE+, B220+) in MLNs from the indicated mice. Saline-treated WT mice: N = 2, *Sting*^*−/−*^ mice: N = 3, HDM-sensitized WT mice: N = 6, and *Sting*^*−/−*^ mice: N = 6. Data are presented as the mean ± SD and representative of three independent experiments. n.s, not significant.

Then, we analyzed B cells in BALF using flow cytometry to elucidate the mechanism underlying decreased IgE levels^34^. The percentage of B cells (B220^+^) in BALF significantly decreased in *Sting*^−/−^ mice compared with WT mice, whereas that of T cells (CD3^+^) did not decrease (Fig. 2A,B). Next, we analyzed IgE-positive B cells in the mediastinal lymph nodes (MLNs) using flow cytometry. Mature B cells differentiate into plasma cells in the MLN^35,36^. The percentage of IgE-positive B cells (B220^+^, IgE^+^) was significantly lower in *Sting*^−/−^ mice than WT mice (Fig. 2C,D). These results suggest that STING is involved in the differentiation of IgE-producing B cells.

### cGAMP, a STING ligand, is involved in IgE production

**Figure 3:**
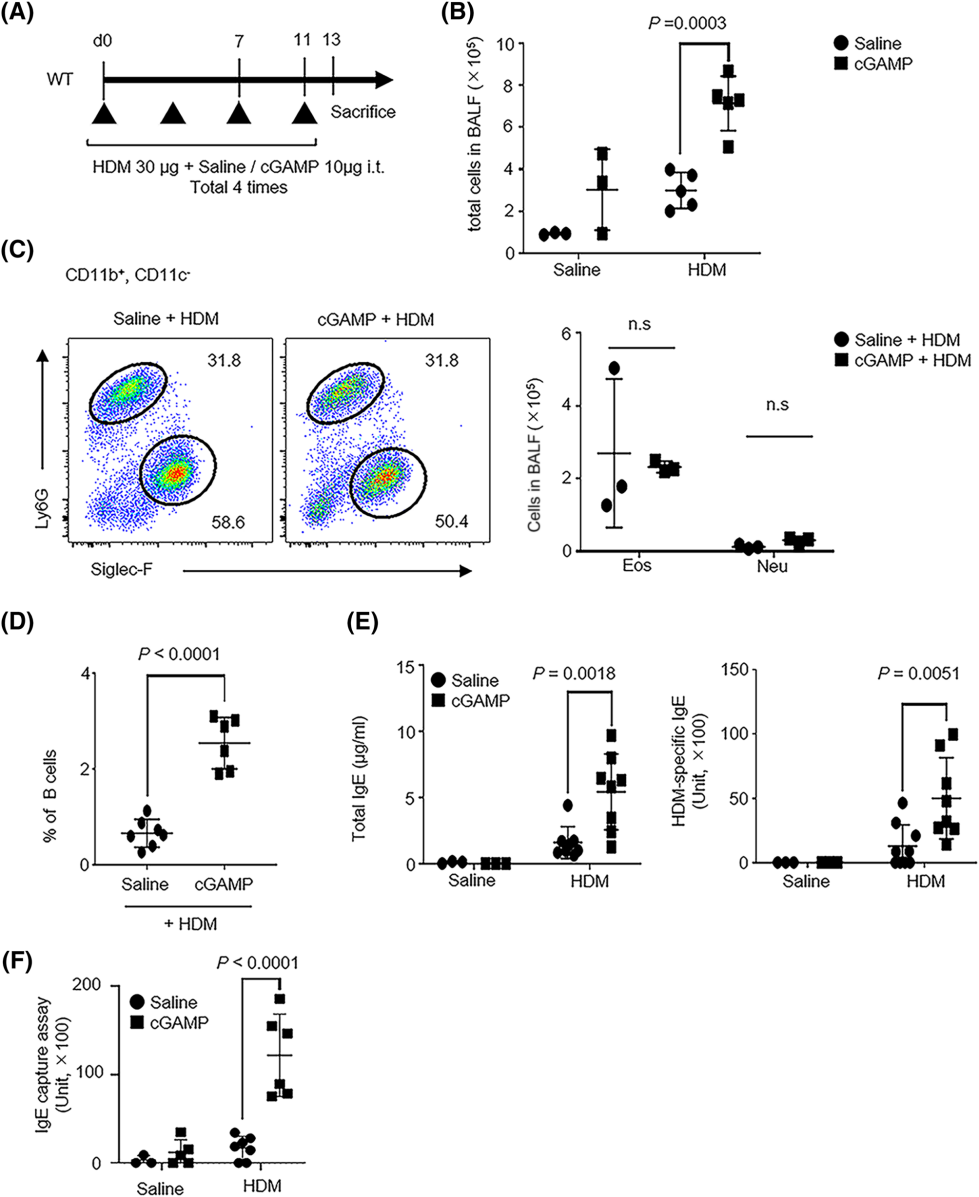
Co-stimulation of HDM and STING ligand increased the serum IgE levels. (**A**) Strategy of HDM- and cGAMP-co-stimulated mice. (**B**) Total number of cells in BALF collected from the indicated mice. Saline + saline-treated mice: N = 3, cGAMP + saline-treated mice: N = 3, saline + HDM-treated mice: N = 5, and cGAMP + HDM-treated mice: N = 5. (**C**, left) Dot plots show the percentage of eosinophils (11b+, 11c−, SiglecF+) and neutrophils (11b+, 11c−, Ly6G+) in BALF from stimulated mice. (**C**, right) These cells were statistically analyzed. Saline + saline-treated mice: N = 3, cGAMP + saline-treated mice: N = 3, and saline + HDM-treated mice: N = 3, and cGAMP + HDM-treated mice: N = 3. (**D**) The percentage of B cells in BALF from stimulated mice. Saline + HDM-treated mice, N = 7 and cGAMP + HDM-treated mice, N = 6. (**E**) Total (left) and HDM-specific IgE levels in serum from stimulated mice. Saline + saline-treated mice: N = 3, cGAMP + saline-treated mice: N = 3, saline + HDM-treated mice: N = 8, and cGAMP + HDM-treated mice: N = 8 for total IgE levels. Saline + saline-treated mice: N = 3, cGAMP + saline-treated mice: N = 3, saline + HDM-treated mice: N = 8, and cGAMP + HDM-treated mice: N = 8 for HDM-specific IgE levels. (**F**) Serum IgE capture assay in the indicated mice. Saline + saline-treated mice: N = 3, cGAMP + saline-treated mice: N = 5, saline + HDM-treated mice: N = 7, and cGAMP + HDM-treated mice: N = 6. Data are presented as the mean ± SD and are representative of three independent experiments. *i.t.* intratracheally, *n.s* not significant, *Neu* neutrophils, *Eo* eosinophils.

Next, to clarify the role of the exogenous direct stimulation of STING in IgE-positive B cells, WT mice were administered cGAMP and HDM extract intratracheally (Fig. 3A). Control mice were administered either saline or cGAMP alone. The number of total cells in BALF increased in the cGAMP + HDM group compared with the HDM alone group (Fig. 3B). Few cells were observed in the saline or cGAMP alone group. When we analyzed the number of eosinophils (11b+, 11c−, SiglecF+) and neutrophils using flow cytometry, no difference was observed between the cGAMP + HDM and HDM alone group (Fig. 3C). In contrast, the proportion of B cells in BALF increased in the cGAMP + HDM group compared with the HDM alone group (Fig. 3D). Total and HDM-specific IgE levels increased the cGAMP + HDM group compared with the HDM alone group (Fig. 3E). In addition to these experiments, we demonstrated serum IgE capture assay. In the cGAMP + HDM group, serum IgE units significantly increased, compared with the HDM alone group (Fig. 3F). These results are comparable to the results of experiments using *Sting*^−/−^ mice.

### Decrease in IL-4 and IL-13 levels in MLN cells of ***Sting***^***−/−***^ mice restimulated by HDM

**Figure 4:**
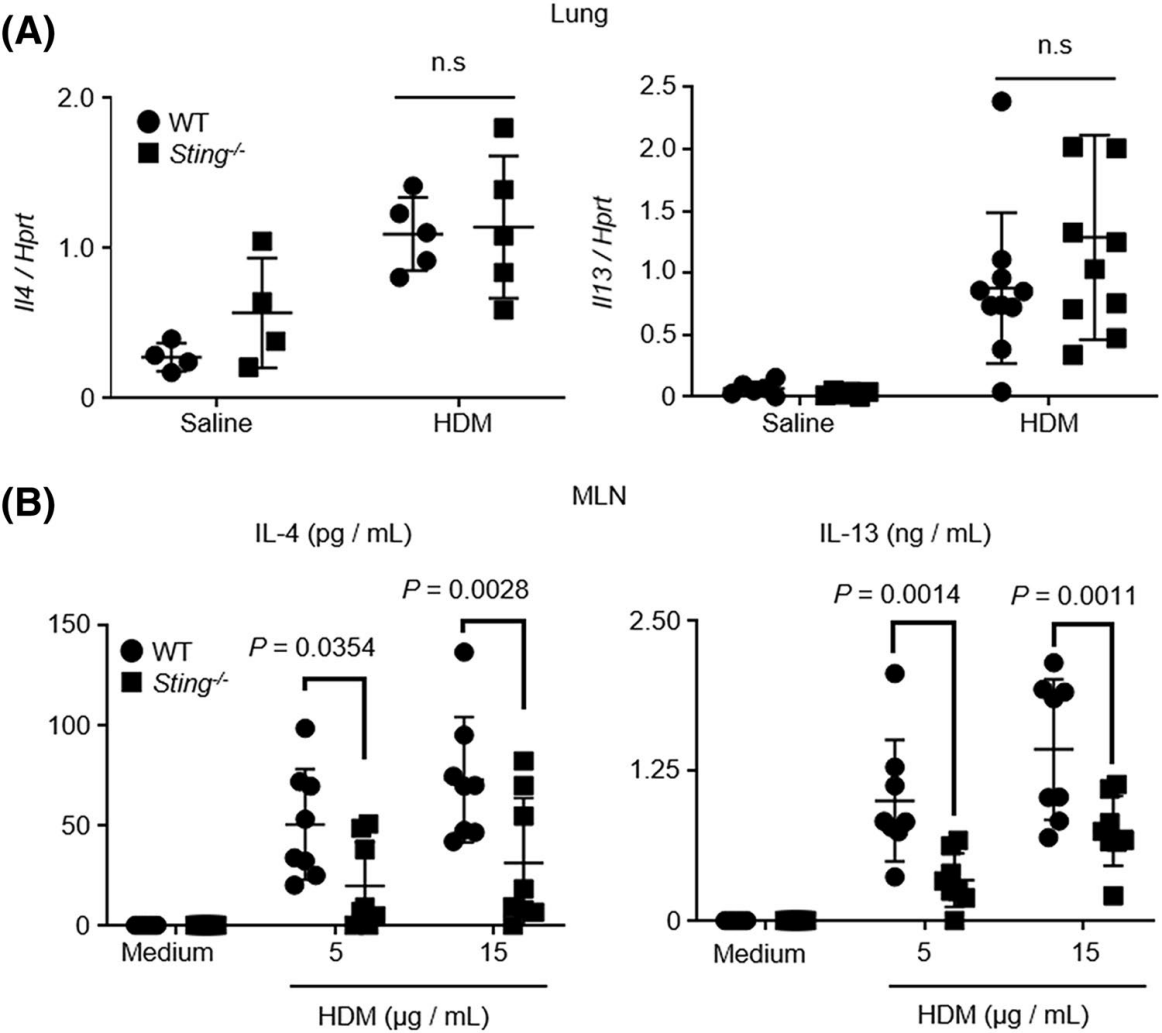
IL-4 and IL-13 production decreased in *Sting*^*−/−*^ MLN cells but not in the lungs. (**A**) Expression levels of *IL-4* and *IL-13* mRNA in the lungs of the indicated mice. For *IL-4*, saline-treated WT mice: N = 4, *Sting*^*−/−*^ mice: N = 4, HDM-sensitized WT mice: N = 5 and *Sting*^*−/−*^ mice: N = 5. For *IL-13*, saline-treated WT mice: N = 6, *Sting*^*−/−*^ mice: N = 6, HDM-sensitized WT mice: N = 10, and *Sting*^*−/−*^ mice: N = 10. (**B**) Mediastinal lymph node cells from the indicated mice were restimulated with 5 or 15 µg/mL HDM. Five days later, IL4 and IL13 levels in the culture supernatant were measured by ELISA. HDM-sensitized WT mice: N = 8, and *Sting*^*−/−*^ mice: N = 8. Data are presented as the mean ± SD and are representative of two independent experiments. *n.s* not significant, *MLN* mediastinal lymph node.

IL-4 and IL-13 are required for IgE production^37–39^. Thus, we analyzed IL-4 and IL-13 in the lungs of HDM-treated WT and *Sting*^*−/−*^ mice using real-time polymerase chain reaction (RT-PCR). However, no differences in the IL-4 and IL-13 levels between WT and *Sting*^*−/−*^ mice were observed (Fig. 4A). To further examine cytokine production at the local site of B cell differentiation, we extracted MLN cells from HDM-induced mice and restimulated the cells with HDM. We then analyzed the levels of IL-4 and IL-13 in the culture supernatant and found that these levels were decreased in *Sting*^*−/−*^ mice compared with WT mice (Fig. 4B).

### Decrease in the number of T follicular helper (Tfh) cells in the MLNs of ***Sting***^−/−^ mice

**Figure 5:**
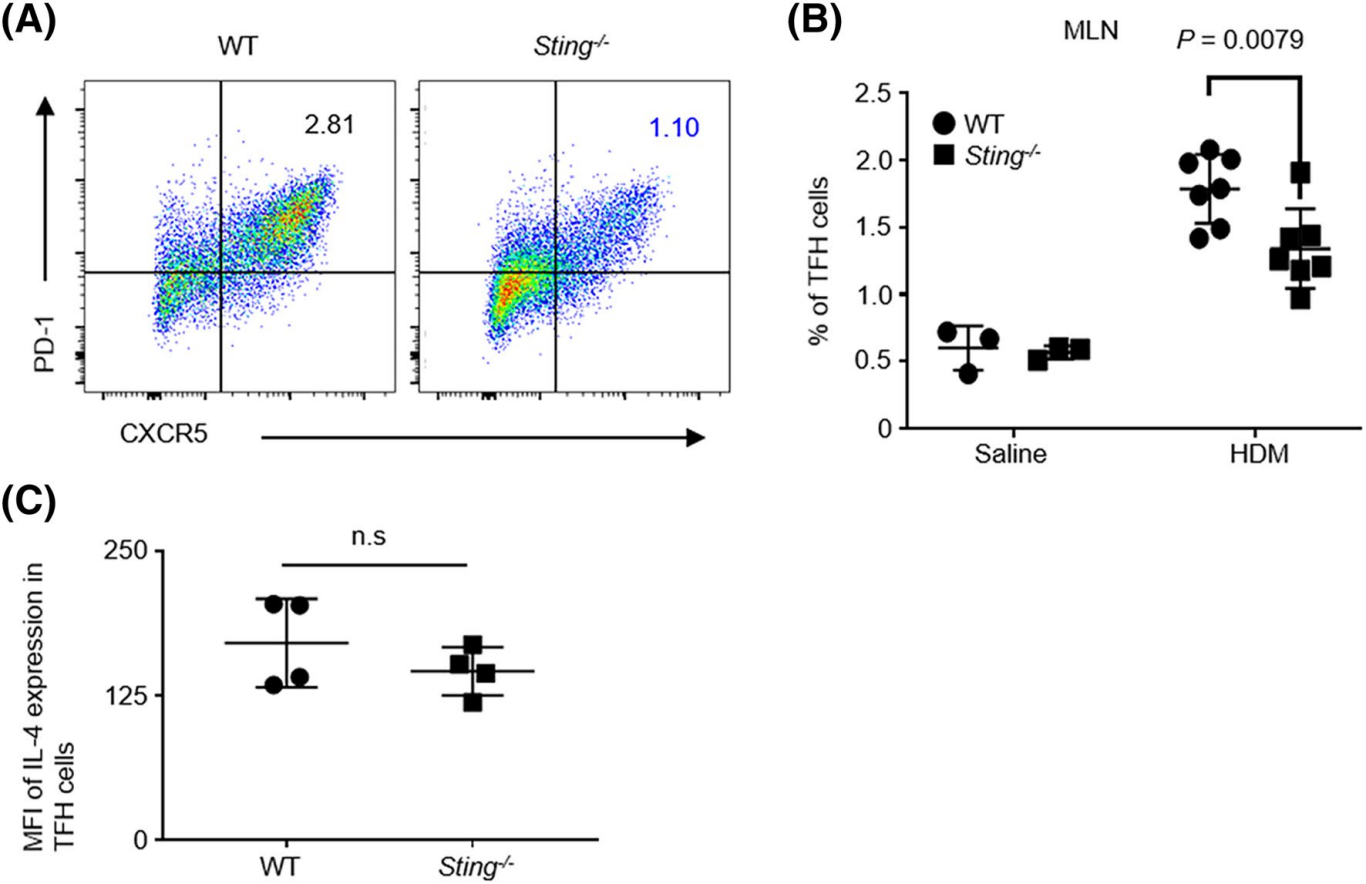
IL-4-producing TFH cells decreased in *Sting*^*−/−*^ mice. (**A**,**B**) The percentage of TFH cells (CD3+, CD4+, CD8α−, CD25−, CD44+, CXCR5+, and PD-1+) in MLNs from the indicated mice. Saline-treated WT mice: N = 3, *Sting*^*−/−*^ mice: N = 3, HDM-sensitized WT mice: N = 7, and *Sting*^*−/−*^ mice: N = 7. (**C**) IL-4 in TFH cells from the indicated mice was intracellularly stained and analyzed by flow cytometry. HDM-sensitized WT mice: N = 4, *Sting*^*−/−*^ mice: N = 4. Data are presented as the mean ± SD and are representative of two independent experiments. *n.s* not significant, *MLN* mediastinal lymph node, *MFI* median fluorescence intensity.

Next, we analyzed Tfh cells in the MLNs (CD3^+^, CD4^+^, CD8α^−^, CD25^−^, CD44^+^, CXCR5^+^, PD-1^+^) of HDM-induced mice using flow cytometry (Fig. 5A). Tfh cells induce the class switching of B cells in the germinal center, such as the spleen or lymph nodes, and promote IgE production by producing IL-4 and IL-13^40–42^. The number of Tfh cells in the MLNs was found to be significantly lower in *Sting*^*−/−*^ mice than WT mice (Fig. 5B). When IL-4 synthesis in Tfh cells was analyzed by intracellular cytokine staining using flow cytometry, IL-4 expression in Tfh cells was not found to differ between WT and *Sting*^*−/−*^ mice (Fig. 5C). These data suggest that STING affects the number, but not the function, of Tfh cells.

## Discussion

In the present study, STING was found to be closely involved in IgE production. The total and HDM-specific serum IgE levels were lower in *Sting*^−/−^ mice than WT mice. In recent years, Tfh cells have been found to play an important role in the differentiation of IgE-positive B cells at the germinal center of the spleen or lymph nodes^40–44^. Therefore, we analyzed MLNs and found that the number of Tfh cells in the MLNs decreased in *Sting*^−/−^ mice. These results suggest that STING may affect Tfh cells and the class-switch recombination of B cells, resulting in defective IgE production.

HDM, a major cause of asthma, directly affects epithelial cells to trigger asthma^45^. HDM acts on various cells including epithelial cell and mast cells and to produce IL-33^46,47^. DNA damage at the site of allergic inflammation, and accumulation of cytosolic dsDNA is demonstrated also shown in airway epithelium treated by allegens^48,49^. Cytosolic dsDNA can activate STING to promote inflammation^8,12,50^. Therefore, we explored the role of STING in pathophysiology of allergic asthma using *Sting*^−/−^ mice. A previous study reports increased production of IgE following the administration of cGAMP^33^. This result is comparable to ours. We further examined its mechanism and found that STING affected B cell differentiation. To elucidate the mechanism underlying IgE production, we focused on IL-4 and IL-13, which are key cytokines for IgE production^37–39^. There were no differences in *IL-4* and *IL-13* levels in the lungs between *Sting*^*−/−*^ and WT mice when stimulated with HDM. Because IL-4 and IL-13 play an essential role in airway inflammation, this result is consistent with the finding that there were no differences in airway inflammation between the two mice. Next, we analyzed the synthesis of IL-4 and IL-13 in the MLNs, where B cells are locally differentiated^38,41^. In addition, we examined the number of Tfh cells and found that it decreased in *Sting*^*−/−*^ mice compared with WT mice. To support our data, another group has shown Tfh cells are less induced by the transfer of autoreactive CD4 + T cells in *Sting*^*−/−*^ mice^51^. These results suggest that STING is involved in the class switching of IgE production through the action of Tfh cells.

STING is expressed in B, T, and dendritic cells (see Supplementary Fig. 1)^15^. STING triggers the direct activation of B cells by regulating B-cell receptor (BCR)^52,53^. To examine the direct effects on B cells, we analyzed B cell proliferation. However, no differences were observed between *Sting*^*−/−*^ and WT mice (see Supplementary Fig. 2B). Because STING has been reported to be dysfunctional in human B cells^54^, our data suggest that Tfh cells are involved in human IgE production.

Airway inflammation following administration of cGAMP is controversial in the HDM-induced asthmatic mouse model. Ozawa et al. have reported an increase in airway inflammation^33^, whereas She et al. a decrease^55^. However, in the present study no difference was observed between the cGAMP + HDM and HDM alone groups. Ozasa et al. has reported the increase in airway inflammation in an IL-33 dependent manner following the administration of cGAMP to the HDM-induced asthmatic mouse mode^33^. Our amount of HDM was greater than that reported (120 µg vs. 5 µg total HDM^33^), because we tried to compare cGAMP-administered WT mice with *Sting*^*−/−*^ mice using the same amount of HDM. High doses of HDM induce severe inflammation^56^, which interfere with the results of cGAMP in allergic inflammation. Furthermore, Han et al. has shown that cGAS, which modifies STING ligand from the DNA of damaged cells, is critical for IL-33 induced airway inflammation via cytosolic-DNA sensing^48^. In contrast to above reports^33,48^, IL-33 induction of our murine HDM-induced model was less although our model possesses phenotype allergic asthma; eosinophilic inflammation, airway hyperreactivity and allergen-specific IgE production^56^. It may be one reason that affect the discrepancy of the results. In contrast to these reports and ours, inhibitory effect of cGAMP has been reported^55^. Pretreatment by cGAMP influences alveolar macrophages and group 2 innate lymphoid cells to suppress IL-33-driven type 2 inflammation^55^. One possible explanation for discrepancy among reports may be the difference of the timing of cGAMP administration; pre or simultaneous treatment with allergen. Further studies are required to clarify the effect of cGMAP.

STING expression is enhanced in the peripheral blood of patients with asthma and involved in the exacerbation of this disease^50^. Furthermore, in IL-33-stimulated human bronchial cells, accumulation of cytosolic dsDNA and cGAS-dependent cytokine production are observed, suggesting allergic inflammation via cytosolic-DNA sensing^48^. In contrast, STING expression in airway epithelial cells is reduced in eosinophilic sinusitis, which induces the enhanced production of IL-13 by epithelial cells^57^. Further studies are required for understanding the role of STING in different human allergic states.

Because we could not identify the effect of STING on airway inflammation, it may be difficult to control allergic states targeting STING alone. However, the new pathway described herein, comprising STING and Tfh cells, provides an alternative approach for the treatment of allergies. Our findings indicate that the endogenous STING ligand affected allergen-specific IgE production via Tfh cells, highlighting a novel mechanism for allergen-specific IgE production.

## Materials and methods

### Study approval

All experiments were approved by the Animal Research Committee (approval no. 05-A-2018, 05-A-2019, 05-A-2020) and the DNA Biosafety Committee (approval no. ExH30-#5, ExH31-#5, ExR01-#5) of Musashino University and performed in accordance with Japan’s and the university’s guidelines.

### Mice

C57BL/6N female mice (8–10 weeks old) were purchased from Sankyo Labo Service Corporation, Inc. (Tokyo, Japan). *Sting*^*−/−*^ mice on a C57BL/6 background were kindly provided by Dr. Glen N. Barber’s laboratory (University of Miami, Miller School of Medicine, Miami, FL, USA)^8^. The mice were housed in a specific-pathogen-free environment with free access to food and water. The care and use of the animals followed the principles of laboratory animal care formulated by Japan and the university’s guidelines.

There were no exclusions of animals, experimental units, or data points reported. Experimental animals were randomly allocated to control and treatment groups. Authors were aware of the group allocation at any stage of the experiment or data analysis. All animal experiments comply with the ARRIVE Essential 10.

### Allergic asthma induction

HDM extract (from *Dermatophagoides farinae*); LPS: 20200 EU/mg and protease: 0.205 Relative activity (0.5 μg HDM/0.01U papain) was kindly provided by the Institute of Tokyo Environmental Allergy (ITEA), Inc. (Tokyo, Japan). Allergic asthma-induced mouse models have already been established, and some modifications have been made to a method that can reproduce the pathophysiology of asthma^56,58,59^. C57BL/6N (WT) or *Sting*^*−/−*^ mice were anesthetized by intraperitoneally administering ketamine (90 mg/kg) and medetomidine (1 mg/kg), following which we intratracheally injected them with 30 µg of HDM or normal saline (Ootsuka, Tokushima, Japan) once every other day, for a total of seven times. Twenty-four hours after the final injection, the HDM-sensitized mice were sacrificed and analyzed. In some experiments, 10 µg cGAMP (Thermo Fisher Scientific, Tokyo, Japan) was administered in addition to HDM or saline.

### Analysis of BALF

BALF was obtained by washing the lungs twice with 1 mL of sterile normal saline under anesthesia and centrifuging the obtained solution at 540×*g* for 5 min at 4 °C. The pellets were treated with red blood cell lysis buffer (Biolegend, San Diego, CA, USA) and resuspended in 1 mL of PBS (−), following which the number of cells were counted. Cytospin preparations were stained with Diff-Quik (International Reagents Corporation, Osaka, Japan) and examined under an optical microscope.

### ELISA

The total IgE and HDM-specific IgE levels were measured in the serum of HDM-sensitized mice. Total IgE levels were measured using an ELISA MAX Deluxe Set (BioLegend). To measure HDM-specific IgE levels, 96-well plates (Greiner Bio One, Kremsmunster, Austria) were coated with 50 µg/mL HDM extract in coating buffer (Bio Rad, Hercules, CA, USA) and incubated overnight at 4 °C. These plates were blocked with Blocking One (Nacalai Tesque, Osaka, Japan) overnight. Next, serum samples were added to these plates and incubated for 2 h at 37 °C. After washing, the cells were treated with biotinylated monoclonal antibody against mouse IgE (YAMASA, Chiba, Japan) and incubated for 1 h at 37 °C. After washing, horseradish peroxidase (HRP)—conjugated streptavidin (BioLegend) was added and incubated for 1 h at 37 °C. The serum of the mice injected intraperitoneally with HDM and Imject Alum Adjuvant (Thermo Fisher Scientific) was used to prepare the standard and diluted with Solution A (Nacalai Tesque). For serum IgE capture assay, 96-well half plates (Greiner Bio One) were coated with 2 µg/mL purified monoclonal antibody against mouse IgE (YAMASA) in coating buffer (Bio Rad) and incubated overnight at 4 °C. These plates were blocked with Blocking One (Nacalai Tesque) for 1 h at 37 °C. Next, serum samples were added to these plates and incubated overnight at 4 °C. After washing, the plates were treated with 10 µg/mL biotinylated HDM extract and incubated for 1 h at 37 °C. After washing, horseradish peroxidase (HRP)—conjugated streptavidin (BioLegend) was added and incubated for 30 min at 37 °C. The serum of the mice injected intraperitoneally with HDM and Imject Alum Adjuvant (Thermo Fisher Scientific) was used to prepare the standard and diluted with Solution A (Nacalai Tesque). IL-4 and IL-13 levels in the supernatant of MLN cells stimulated by HDM (detailed below) were measured using a mouse IL-4 Quantikine ELISA kit (R&D systems, Minneapolis, USA) and mouse IL-13 DuoSet ELISA (R&D Systems). A plate reader (TECAN, Mannedorf, Switzerland) was used to measure the absorbance of each well at 450 nm.

### Reagents and antibodies

Anti-mouse CD16/32 (clone: 93), CD3-PE-Cy7 (clone: 17A2), CD4-BV510 (clone: RM4-5), CD8α-Percp/Cy5. 5 (clone: 53-6. 7), B220-FITC (clone: RA3-6B2), B220-APC (clone: RA3-6B2), IgE-FITC (clone: RME-1), CD11b-APC (clone: M1/70), Ly-6G-PE (clone 1A8), Ly-6C- Percp/Cy5. 5 (clone: HK1. 4), CD11c-BV421 (clone: N418), Siglec-F-FITC (clone: S17007L), CD25-PE (clone: PC61. 5), CD44-APC-Cy7 (clone: IM7), PD1-FITC (clone: 29F. 1A12), CXCR5-BV421 (clone: L138D7), and IL4-APC (clone: 11B11) antibodies were purchased from BioLegend. Anti-STING (clone: 41) antibody, DNAse I from bovine pancreas, and collagenase D were purchased from Sigma-Aldrich (Tokyo, Japan).

### Cell culture

MLN cells were cultured in RPMI 1640 complete medium: Roswell Park Memorial Institute (RPMI) 1640 medium (Nacalai Tesque) supplemented with 10% fetal bovine serum (BioWest, Kansas City, KS, USA), antibiotic–antimycotic mixed stock solution (100×) from Nacalai Tesque, and 50 μM 2- mercaptoethanol from Wako (Tokyo, Japan).

### Flow cytometry

A single-cell suspension was prepared for staining infiltrating cells in BALF and MLNs from HDM-sensitized mice. Red blood cells in the specimens were lysed in RBC lysis buffer (BioLegend). The Fc receptor on the prepared cells was blocked with anti-CD16/32 antibody. The cells were then counterstained. Staining for B220, CD3, CD4, and CD8α was conducted to separate B cell and T cell subsets. Staining for B220, CD11b, CD11c, and anti-mouse IgE antibodies was conducted to separate IgE-positive B cells. Staining for CD11b, CD11c, Ly6G, and Siglec-F was conducted to separate neutrophils and eosinophils. Staining for CD3, CD4, CD8α, CD25, CD44, CXCR5, and PD1 was conducted to separate Tfh cells. To stain STING, the cells were treated with fixation/permeabilization buffer (BD Biosciences, Franklin Lakes, NJ, USA) for 20 min at 4 °C after counterstaining with each cell marker. The cells were washed twice with 1 × Perm/Wash buffer (BD Biosciences) and incubated in 2 µg/mL anti-STING antibody in 1 × Perm/Wash buffer for 30 min at 4 °C. Next, the cells were incubated with 0.2 µg/mL mouse anti-rat IgM for 30 min at 4 °C. They were then suspended in staining buffer (1 × PBS (−) containing 0.1% sodium azide and 10% fetal calf serum (FCS)). To stain IL-4, MLN cells from HDM-sensitized mice were cultured 4 h in RPMI1640 complete medium with 100 ng/mL phorbol myristate acetate (PMA, FUJIFILM Wako pure chemical co.), 1 µg/mL ionomycin (FUJIFILM Wako pure chemical co.) and 10 µg/mL brefeldin A (BD biosciences). After the incubation, MLN cells were stained with CD3, CD4, CD8α, CD25, CD44, CXCR5, and PD1 abs for the analysis of Tfh cells. After counterstaining, the cells were fixed with Fixation/Permeabilization buffer (BD Biosciences, Franklin Lakes, NJ, USA) for 20 min at 4 °C. The cells were then washed twice with 1 × Perm/Wash buffer (BD Biosciences) and incubated in 2 µg/mL of anti-IL-4 for 30 min at 4 °C. Finally, the cells were washed twice with 1 × Perm/Wash buffer and suspended in a staining buffer (10% FCS, 0.1% Sodium azide in 1 × PBS) for flow cytometric analysis by FACS Lyric (BD Biosciences). Flow cytometry data were analyzed using FlowJo software (Ashland, OR, USA).

### RNA extraction and complementary DNA (cDNA) synthesis

RNA was extracted as reported in our previous study^27^. Briefly, the lungs from HDM- or normal-saline-treated mice were incubated with Sepasol-RNA I Super G solution for RNA isolation (Nacalai Tesque, Osaka, Japan) and homogenized with metal beads in a multi-bead shocker (Yasui Kikai, Osaka, Japan). Then, the crushed samples were added to 200 μL of chloroform and centrifuged at 15,300×*g* at 4 °C for 15 min. The supernatant was collected, and 500 μL of isopropanol was added. The sample was then mixed well, and the mixture was centrifuged at 15,300×*g* at 4 °C for 10 min. The supernatant was discarded, and 70% ethanol was added to the nucleic acid pellets. The pellet was then centrifuged at 15,300×*g* at 4 °C for 5 min. The supernatant was discarded, and the nucleic acid pellet was dried and dissolved in 200 μL of water.

cDNA was synthesized from the extracted RNA using ReverTra Ace qPCR Master Mix (Toyobo, Osaka, Japan).

### RT-PCR

To measure the mRNA levels, cDNA was quantified by RT-PCR using TaqMan probes and primers (indicated below) (Sigma-Aldrich Japan, Meguro, Japan) in a total volume of 20 μL. The expression of genes of interest was calculated according to the comparative threshold cycle method, using the hypoxanthine–guanine phosphoribosyl transferase gene (*Hprt*) as an internal control.

*Il4* probe, 5′-TCCTCACAGCAACGAAGAACACCACAGA-3′

*Il4* forward, 5′-CACGGAGATGGATGTGCCAAA-3′

*Il4* reverse, 5′-GCGAAGCACCTTGGAAGCC-3′

*Il13* probe, 5′-GCCGGTGCCAAGATCTGTGTCTCTCCCT-3′

*Il13* forward, 5′-TGGCTCTTGCTTGCCTTGG-3′

*Il13* reverse, 5′-GTTGCACAGGGGAGTCTGG-3′

*Hprt* probe, 5′-AGCTTGCTGGTGAAAAGGACCTCTCGAAGT-3′

*Hprt* forward, 5′-CAGCCCCAAAATGGTTAAGGTTG-3′

*Hprt* reverse, 5′-CCAACAAAGTCTGGCCTGTATCC-3′

### Restimulation of MLN cells

MLN cells (2 × 10^5^) from HDM-sensitized mice were seeded on 96 well plates and restimulated with 5 or 15 μg/mL HDM. Five days after incubation, IL-13 and IL-4 levels in the culture supernatants were quantified by ELISA.

### Statistical analysis

Statistical significance was calculated using two-tailed unpaired Student’s t test or two-way ANOVA. Statistical analyses were performed using Prism8 (GraphPad Software, San Diego, USA). Statistical significance was set at P < 0.05.

## Supplementary Information

Supplementary Information 1.
