# Supplementary material for: Crucial role of stimulator of interferon genes-dependent signaling in house dust mite extract-induced IgE production
Source: Sci Rep. 2021 Jun 23;11:13157. doi: 10.1038/s41598-021-92561-w (PMC8222396; doi:10.1038/s41598-021-92561-w)
Supplement: Supplementary file 1 — Supplementary Information 1. [file 41598_2021_92561_MOESM1_ESM.pdf]

Title: Crucial role of stimulator of interferon genes-dependent signaling in house dust mite extract-induced IgE production

Authors: Hiroki Nunokawa[1,2], Yusuke Murakami[1,3], Takashi Ishii[1], Tomoya Narita[1,3], Haruyuki Ishii[2], Hajime Takizawa[2], Naomi Yamashita[1,3]

Affiliations:

1, Research Institute of Pharmaceutical Sciences, Musashino University, Nishitokyo-shi, Tokyo, Japan

2, Department of Respiratory Medicine, Kyorin University School of Medicine, Mitaka-shi, Tokyo, Japan

3. Faculty of Pharmacy, Department of Pharmaceutical Sciences, Musashino University, Nishitokyo-shi, Tokyo, Japan

Corresponding author: Naomi Yamashita, MD, PhD,

Research Institute of Pharmaceutical Sciences, Musashino University, Nishitokyo-shi, Tokyo, 202-8585, Japan.

Tel: +81-42-468-8647

Email: naoyama@musashino-u.ac.jp

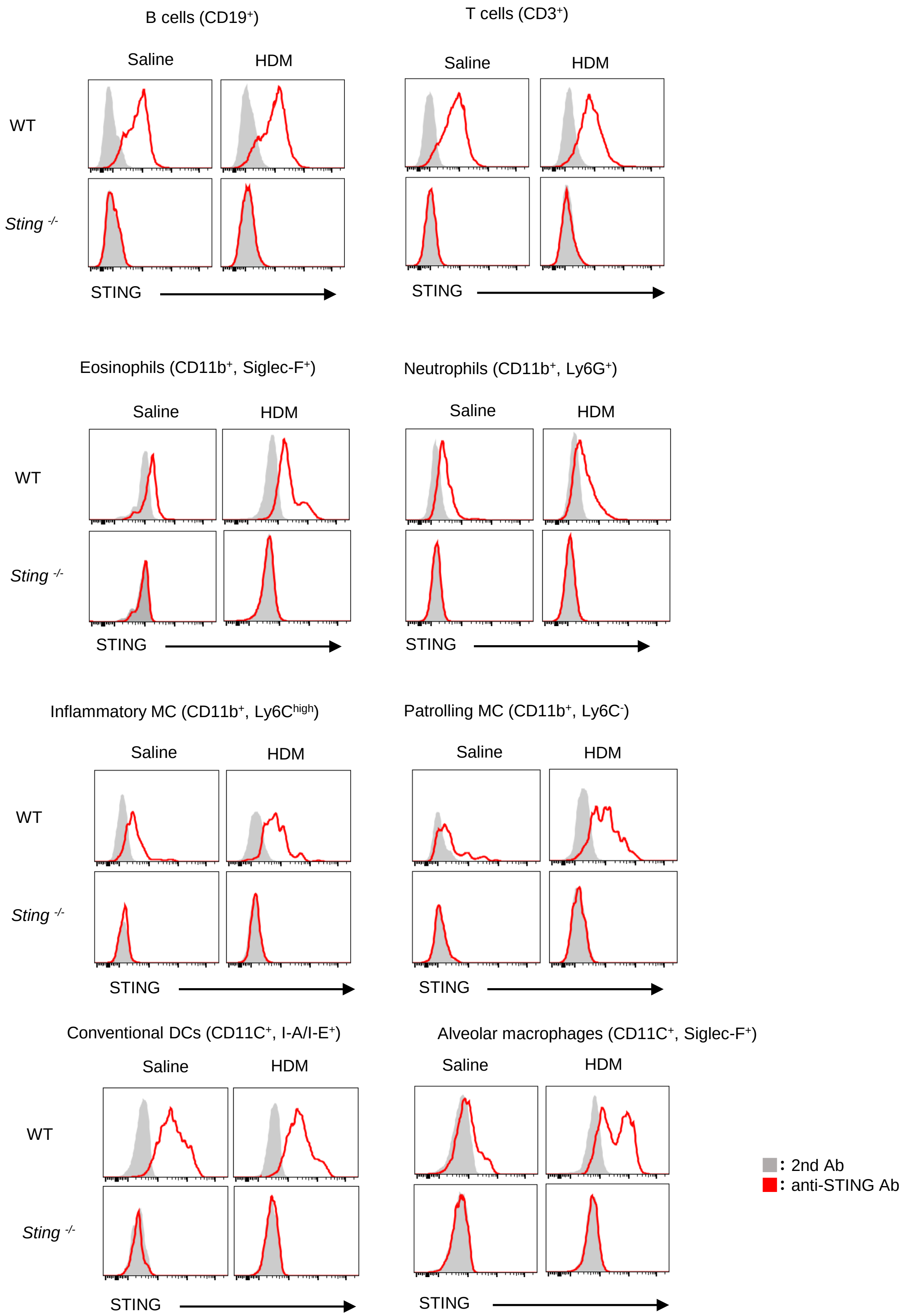

Figure S1. STING expression in lungs

Flow cytometry analysis of STING expression in lungs from WT and *Sting* $^{-/-}$ mice. B cells, T cells, eosinophils, neutrophils, monocyte subsets, conventional DCs and alveolar macrophages were stained for the markers indicated and analyzed. Gray histograms show 2nd reagent only. Red lines show anti-STING antibody staining. Data are presented from twice independent experiments (n = 2 each). DCs: Dendritic cells, MCs: Monocytes

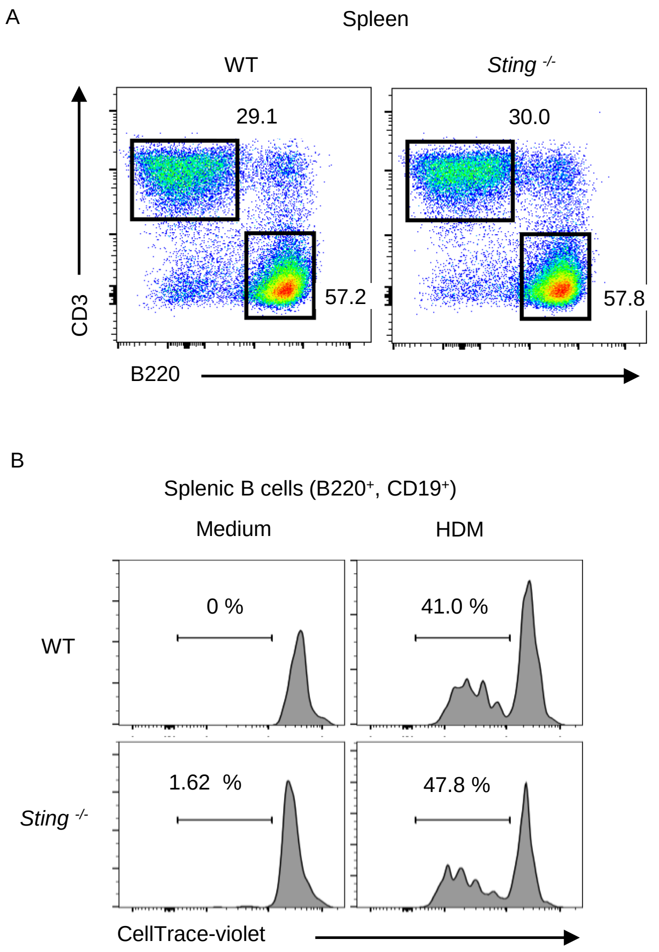

Figure S2. Flow cytometry analysis of B cell proliferation in response to HDM

A, Splenic B cells and T cells were stained for the markers indicated and analyzed. B, Splenic B cells were sorted by indicated markers and treated with the dye. After that, B cells were stimulated with HDM for 3 days and analyzed. The percentages show proliferation level. Data are presented from twice independent experiments (n = 3 each).
